# Identification and use of the sugarcane bacilliform virus enhancer in transgenic maize

**DOI:** 10.1186/s12870-014-0359-3

**Published:** 2014-12-19

**Authors:** John P Davies, Vaka Reddy, Xing L Liu, Avutu S Reddy, William Michael Ainley, Mark Thompson, Lakshmi Sastry-Dent, Zehui Cao, James Connell, Delkin O Gonzalez, Douglas Ry Wagner

**Affiliations:** Dow AgroSciences, 16160 SW Upper Boones Ferry Rd, Portland, OR 97224 USA; Dow AgroSciences, 9330 Zionsville Rd, Indianapolis, IN 46268 USA; Current address: GEVO, Inc., 345 Inverness Dr S C-310, Englewood, CO 80112 USA; Current address: Agrinos, Inc, 279 Cousteau Place, Davis, CA 95618 USA

**Keywords:** Promoter, Enhancer, Transcription, Transgenic plant, Transient assay

## Abstract

**Background:**

Transcriptional enhancers are able to increase transcription from heterologous promoters when placed upstream, downstream and in either orientation, relative to the promoter. Transcriptional enhancers have been used to enhance expression of specific promoters in transgenic plants and in activation tagging studies to help elucidate gene function.

**Results:**

A transcriptional enhancer from the Sugarcane Bacilliform Virus - Ireng Maleng isolate (SCBV-IM) that can cause increased transcription when integrated into the the genome near maize genes has been identified. In transgenic maize, the SCBV-IM promoter was shown to be comparable in strength to the maize ubiquitin 1 promoter in young leaf and root tissues. The promoter was dissected to identify sequences that confer high activity in transient assays. Enhancer sequences were identified and shown to increase the activity of a heterologous truncated promoter. These enhancer sequences were shown to be more active when arrayed in 4 copy arrays than in 1 or 2 copy arrays. When the enhancer array was transformed into maize plants it caused an increase in accumulation of transcripts of genes near the site of integration in the genome.

**Conclusions:**

The SCBV-IM enhancer can activate transcription upstream or downstream of genes and in either orientation. It may be a useful tool to activate enhance from specific promoters or in activation tagging.

## Background

Enhancers are DNA elements that are able to increase transcription from other promoters whether they are placed upstream or downstream of transcription start sites and their promoter enhancing activity is independent of orientation relative to the transcription start site [1,2]. Enhancers that are effective in plants have been isolated from genes of plants as well as from genes of viruses and bacteria that infect plants. These include enhancers from the tobacco tCUP [3,4], the pea plastocyanin [5], the Cauliflower mosaic virus 35S (CaMV 35S) [6,7], the Figwort mosaic virus [8] and the *Agrobacterium tumefacians* 780 [9,10] and *ocs* promoters [11].

Plant virus-derived promoters have been shown to be a rich source of strong constitutive promoters for use in plant biology and several have been shown to contain enhancer sequences [6,8]. The CaMV 35S promoter has been used extensively in driving transgenes in transgenic plants. Many other viral promoters have also been shown to effectively drive expression of transgenes; CaMV 19S, Rice tungro bacilform virus (RTBV) [12], Soybean chlorotic mottle virus [13], Mirabilis mosaic virus [14,15], Figwort mosaic virus (FMV) [16,17], Peanut streak chlorotic virus [18], Banana streak badnavirus [19], Cestrum yellow leaf curling virus (CmYLCV) [20] and Sugarcane bacilliform badnavirus (SCBV) [19,21,22]. Among these, the CaMV 35S and the FMV promoters have been demonstrated to have enhancer sequences within the promoter [6,8].

The CaMV 35S enhancer [7] is the most common enhancer used in plant biology. Several studies have shown that the CaMV 35S promoter is not as active as other strong constitutive promoters in monocots [23-26], raising the question whether the CaMV 35S enhancer sequences are as effective in monocots as they are in dicots. However, 2x and 4x arrays of the CaMV 35S enhancer have been shown to enhance transcription of heterologous promoters in stable transformants of rice as well as to cause increased transcript accumulation of endogenous genes [27-30].

The Sugarcane bacilliform virus (SCBV), like the Cauliflower mosaic virus, is in the Caulimoviridae family of viruses. While the Cauliflower mosaic virus is in the Caulimovirus genus and mostly infects dicots, SCBV is in the Badnavirus genus along with Commelina yellow mottle virus (CoMV) and RTBV and infects monocots [21]. Badnaviruses have circular genomes that produce a terminally redundant transcript [31,32]. Like CoMV, SCBV has three large open reading frames on its plus strand [33]. Promoters from several Badnaviruses have been shown to drive expression of heterologous genes in transgenic plants [16,19,22,34,35]. Because these viruses infect monocots, they may be useful sources of strong promoters for monocots. SCBV promoters from several isolates of the virus have been tested in transgenic plants and shown to be highly expressed in most tissues tested [21,22,36].

We present a characterization of an 839 bp fragment of the Sugarcane Bacilliform Virus - Ireng Maleng isolate (SCBV-IM) promoter and demonstrate that it is comparable in strength to the strong maize ubiquitin 1 (ZmUBI1) promoter in transgenic maize. This work also presents a dissection of the SCBV-IM promoter and the identification of sequences that can enhance transcription when placed upstream of a truncated maize alcohol dehydrogenase (ZmADH1) promoter. Similar to what was seen with the CaMV 35S enhancer [8,37-39], multiple tandem copies of the SCBV-IM enhancer are more effective in increasing transcription than a single copy. An activation tagging element containing four tandem copies of the enhancer element has been introduced into maize. Examination of events containing the activation tagging element indicates that the 4x SCBV-IM enhancer is capable of causing an increase in accumulation of transcripts of native maize genes near the site of insertion of the SCBV-IM enhancer.

## Results

### The SCBV-IM promoter is a strong promoter in transgenic maize

**Figure 1:**
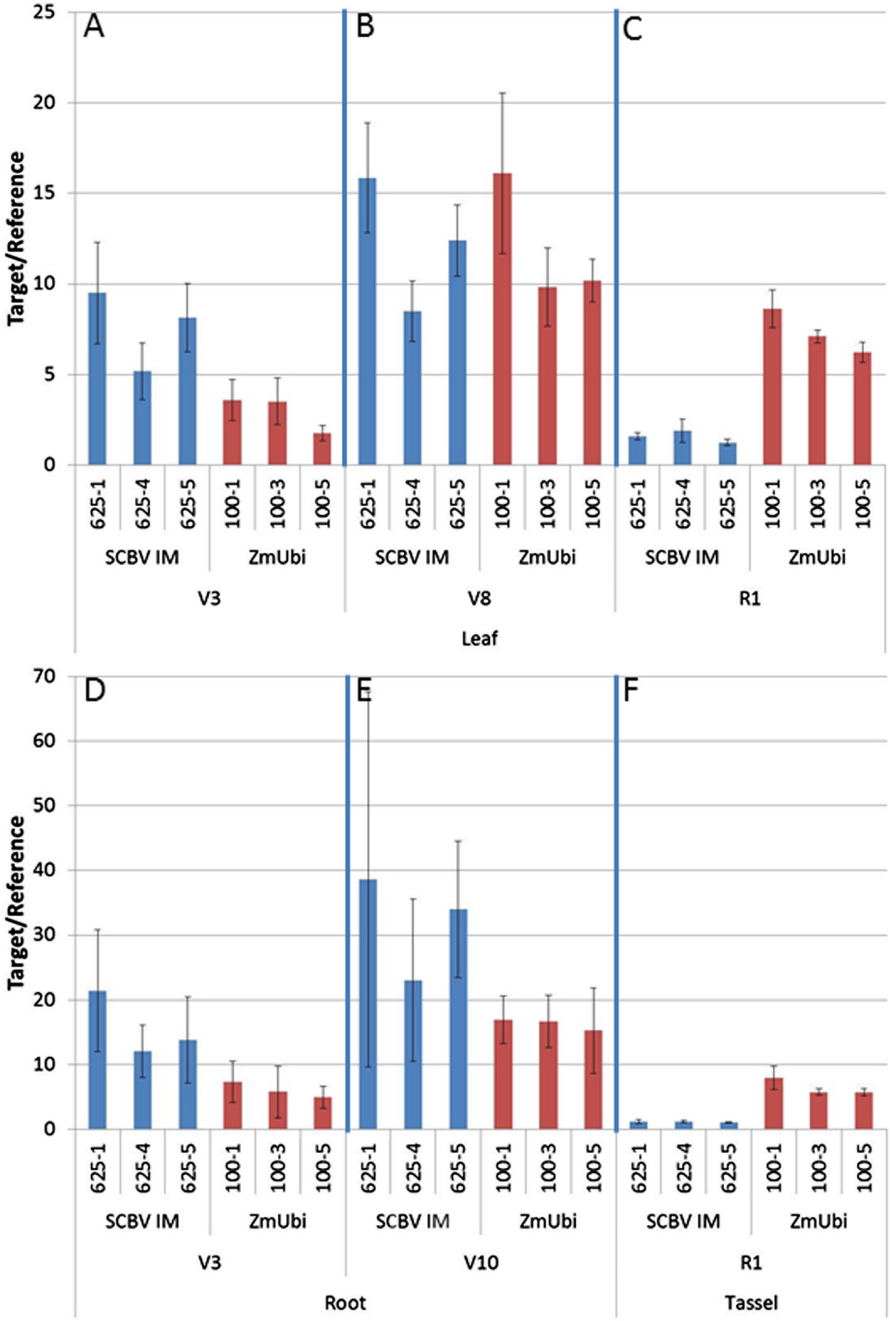
**Comparison of the SCBV-IM and ZmUBI1 promoters in transgenic maize tissues.** Transgenic, single copy maize events containing SCBV-IM::AAD1 and ZmUBI1::AAD1 constructs were analyzed for AAD1 transcript accumulation in the youngest fully expanded leaf of V3 **(A)** and V8 **(B)** stage plants, the leaf just below the ear of R1 plants **(C)**, a 1 cm section from the tip of the root in V3 **(D)** and V10 **(E)** stage plants, and in the tassels of R1 stage plants **(F)**. Three events for each of the two constructs were compared by RT-qPCR using primers specific to the AAD1 transcript and normalized to an endogenous transcript, TIP (for leaf and tassel tissues) or MAZ95 (for root tissues). The error bars represent the standard deviation of three measurements. Analysis of Variance (α = 0.05) indicate that more AAD1 transcript accumulate in the SCBV-IM::AAD1 events than in ZmUBI1::AAD1 events in leaves of V3 stage plants, the roots of V3 and V8 stage plants, while similar levels of AAD1 transcript accumulate in leaves of V8 stage plants. ZmUBI1::AAD1 transgenic plants accumulate more AAD1 transcript than SCBV-IM::AAD1 transgenic plants in leaf and tassel tissues at R1.

To compare the strength of the SCBV-IM promoter relative to a known strong promoter, transgenic plants containing SCBV-IM::AAD1 and ZmUBI1::AAD1 transgenes were generated. AAD1 encodes an enzyme that degrades 2,4-D and aryloxyphenoxypropionate herbicides and plants expressing this gene are tolerant of these herbicides [40]. Transcript accumulation was measured in samples from various tissues of T1 plants by RT-qPCR. Tissue samples were taken from the youngest fully expanded leaf at the V3, and V8 stages, from the leaf below the developing ear at the R1 stage, from a 1 cm section from the tip of a root at V3 and V10 stages and from the tassels at R1 stage. Figure 1 shows AAD1 transcript accumulation in 3 events containing each transgene. In the leaf samples collected at V3 stage, plants containing the SCBV-IM::AAD1 transgene accumulate more transcript than plants containing ZmUBI1::AAD1 (Figure 1A), at the V8 stage, leaves accumulate similar amounts of AAD1 transcripts (Figure 1B) while in the leaf samples of R1 plants lower levels of AAD1 transcript accumulate in the SCBV-IM::AAD1 transgenic plants (Figure 1C). In the roots of V3 and V10 plants, SCBV-IM::AAD1 transgenic plants accumulate more of the AAD1 transcripts than ZmUBI1::AAD1 transgenic plants (Figure 1D and E). In tassel tissues, ZmUBI1::AAD1 transgenic plants accumulate more AAD1 transcript. These results demonstrate that the SCBV-IM promoter is stronger or comprable in strength to the strong, constitutively expressed maize ubiquitin 1 promoter [26] in young leaf and root tissues, but is weaker in the leaf below the devloping ear and in tassel tissues at R1.

### SCBV-IM enhancer identification and characterization

**Figure 2:**
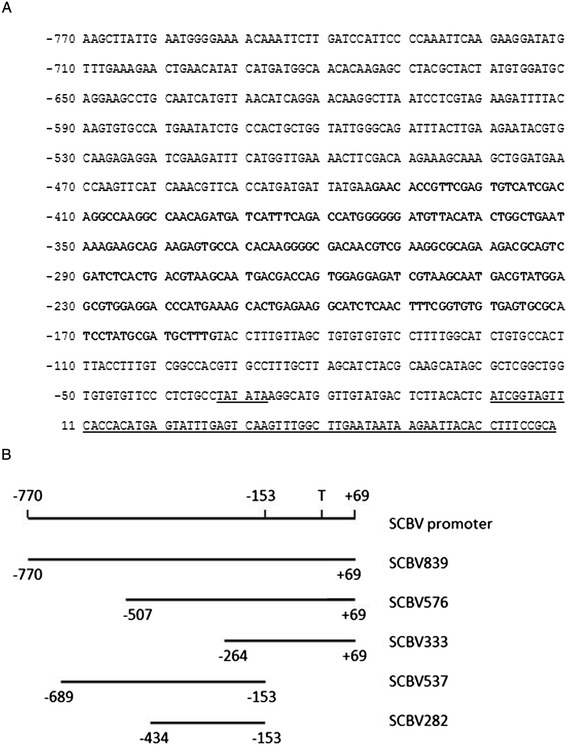
**SCBV promoter sequence (A) and fragments used in experiments (B). (A)** The transcription start site was mapped by 5′ RACE and sequences in the 5′ untranslated region of the transcript are underlined (position 1 – 69). The putative TATA box is underlined at position −28 – -33. The SCBV-IM enhancer sequences are in bold. **(B)** The fragments of the SCBV-IM promoter used in transient assays are displayed. The position of the transcription start site (T) is shown.

The activity of the SCBV-IM enhancer was demonstrated in transient assays first by identifying sequences that are necessary for high levels of transcription and then by identifying sequences that can enhance transcription from a heterologous promoter. The sequence of the SCBV-IM promoter is shown in Figure 2; the transcription start site was mapped by 5′ RACE.

SCBV-IM promoter fragments SCBV839, SCBV576 and SCBV333 (Figure 2) were cloned upstream of the luciferase (LUC) reporter gene. Transcriptional activities of these constructs were tested by transfecting maize Hi-II suspension cells and monitoring relative activities of the reporter genes.

**Figure 3:**
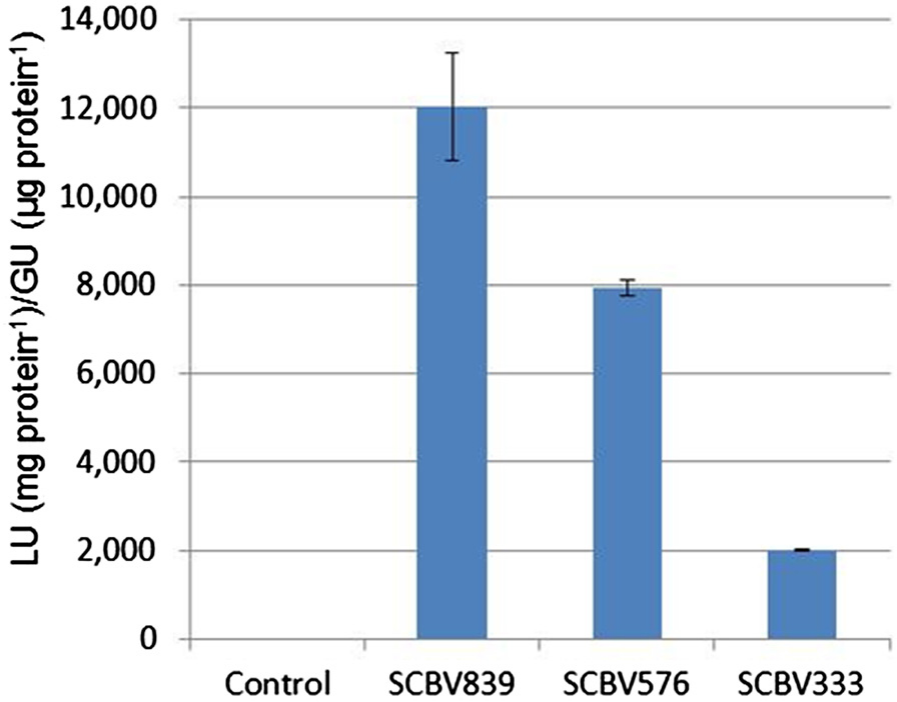
**Promoter analysis of the SCBV-IM promoter.** Three fragments of the SCBV-IM promoter fused with the luciferase reporter gene and tested for activity in transient assays and compared with a luciferase construct without a promoter. These constructs were transfected into maize Hi-II cultures along with a ZmUBI1::GUS construct. Activity is reported as the ratio of Luciferase activity (LU) from the test construct to GUS activity (GU) from the internal control construct. Error bars represent the standard deviation of three measurements. Analysis of Variance and Tukey-Kramer HSD tests (α = 0.05) indicate significant differences in LU/GU ratios for all constructs tested.

To test the activities of different SCBV-IM promoter fragments, equal molar concentrations of the test plasmids and a reference plasmid (ZmUBI:GUS), used as an internal control to normalize for differences in transformation efficiency, were co-introduced into maize Hi-II suspension cell cultures by particle bombardment. Two days after the bombardment, total protein was isolated from transfected cells and LUC and GUS enzymatic activities were determined. Activity was expressed as the ratio of LUC to GUS activity. The results show that the promoter fragment SCBV576 had 60% of the activity of the SCBV839 promoter fragment (Figure 3) and the SCBV333 promoter fragment had only 10% of the activity of the SCBV839 fragment. These data indicate that sequences necessary for most of the SCBV-IM promoter activity reside upstream of the 333 bp fragment.

Next, two upstream fragments of the SCBV-IM promoter were tested for their ability to enhance transcription from a truncated heterologous promoter. Two fragments of the SCBV-IM promoter (SCBV282 consisting of sequences −434 bp to −153 bp and SCBV537 consisting of sequences from −689 to −153, relative to the transcription start site) were cloned upstream of a truncated maize alcohol dehydrogenase 1 (ZmADH1) promoter (−100 to +106, relative to the transcription start site) [41] fused to the firefly luciferase gene. These constructs were designated SCBV282::ZmADH1::LUC and SCBV537::ZmADH1::LUC, respectively.

**Figure 4:**
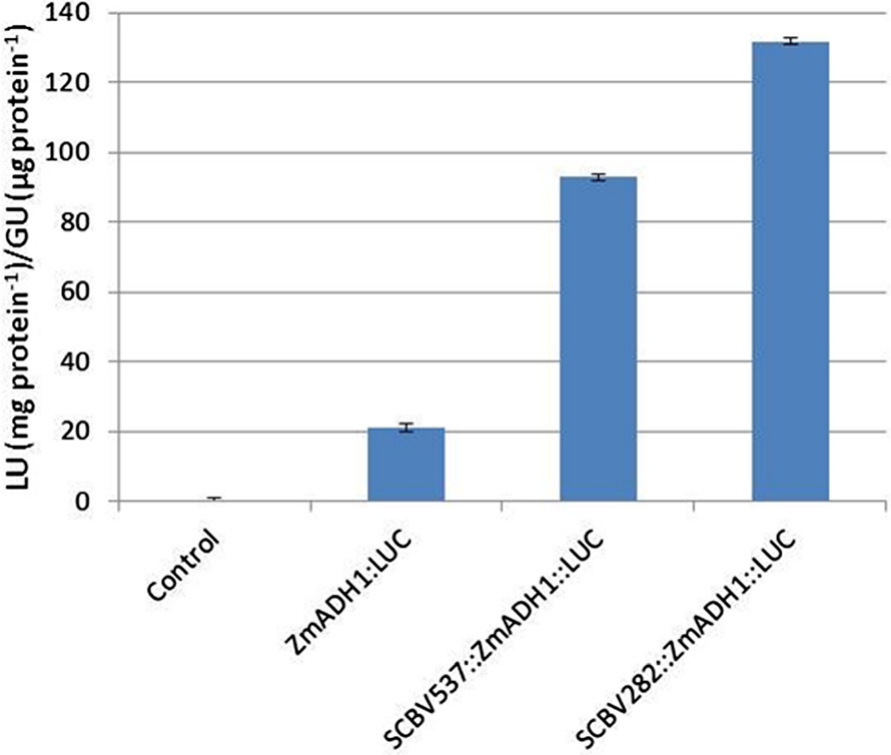
**Enhancer analysis of the SCBV-IM promoter.** Two fragments of the SCBV-IM promoter were fused with the truncated ZmADH1 promoter and the luciferase reporter gene. These constructs were tested for activity in transient assays and compared with a construct containing only the minimal ZmADH1 promoter. These constructs were transfected into maize Hi-II cultures with a ZmUBI1::GUS construct. Activity is reported as the ratio of luciferase activity (LU) from the test construct to GUS activity (GU) from the internal control construct. Error bars represent the standard deviation of three measurements. Analysis of Variance and Tukey-Kramer HSD tests (α = 0.05) indicate significant differences in LU/GU ratios for all constructs tested.

Maize Hi-II suspension cells were transfected with plasmids SCBV282::ZmADH1::LUC, SCBV537::ZmADH1::LUC and ZmADH1::LUC (with no SCBV-IM sequences) along with the reference plasmid containing ZmUBI1::GUS. Overall, these promoters gave much lower activity than the intact SCBV promoter (Figure 4). This may be because the ZmADH1 chimeric promoters are inherently weaker than the intact SCBV-IM promoter or because a promoter fragment of the SCBV-IM promoter necessary for high activity was not included in the chimeric constructs.

The results shown in Figure 4 indicate that the SCBV282 fragment (containing sequence from −434 bp to −153 bp) was able to enhance activity of the truncated ZmADH1 promoter more effectively than the larger SCBV537 fragment (containing sequences from −689 bp to −153 bp). These results indicate that these fragments of the SCBV-IM promoter cause an increase in activity of the reporter gene driven by a truncated heterologous promoter, and that most of this enhancing activity lies within the −434 bp to −153 bp region.

**Figure 5:**
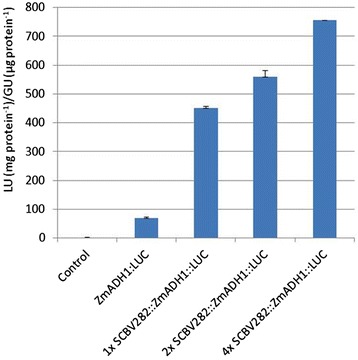
**Analysis of SCBV-IM enhancer arrays.** Tandem copies (1×, 2× and 4×) of the SCBV-IM enhancer were fused with the truncated ZmADH1 promoter and the luciferase reporter gene. These constructs were tested for activity in transient assays and compared with a construct containing only the minimal ZmADH1 promoter. These constructs were transfected into maize Hi-II cultures with a ZmUBI1::GUS construct. Activity is reported as the ratio of Luciferase activity (LU) from the test construct to GUS activity (GU) from the internal control construct. Error bars represent the standard deviation of three measurements. Analysis of Variance and Tukey-Kramer HSD tests (α = 0.05) indicate significant differences in LU/GU ratios for all constructs tested.

To determine whether multiple copies of the SCBV-IM enhancer are more effective in activating transcription, 1, 2 and 4 copies of the SCBV282 fragment were cloned upstream of the truncated ZmADH1 promoter fused to the LUC gene and bombarded into maize Hi-II suspension cells. Constructs containing 1, 2 and 4 tandem copies of the SCBV-IM enhancer had 5 times, 6 times and 10 times more activity, respectively, than did cells bombarded with the ZmADH1::LUC construct without any SCBV-IM sequences (Figure 5). It should be noted that in the experiment shown in Figure 5, the activity of SCBV282::ZmADH1::LUC and ZmADH1::LUC was substantially greater than in the experiment shown in Figure 4. However, similar variation in promoter activity between independent experiments conducted with different cell preparations has previously been reported [42].

### SCBV-enhancer activity in stable maize transformants

**Figure 6:**
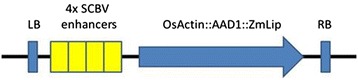
**Schematic of enhancer construct, pEPS1027, used in maize transformation.** The construct includes an array of 4 copies of the SCBV-IM enhancers (yellow boxes) and the AAD1 herbicide resistance gene (blue arrow) containing the rice actin promoter fused with the AAD1 gene and the maize lipase 3′ UTR relative to the left border (LB) and right border (RB) of *Agrobacterium* T-DNA.

To determine whether the SCBV-IM enhancer can increase expression of genes within the maize genome, an activation tagging construct consisting of the 4x tandem array of the SCBV282 enhancer (Figure 6) and a selectable marker composed of the rice actin promoter driving expression of the AAD1 herbicide resistance gene and the maize lipase 3′ UTR [43] was cloned and transformed into maize plants via *Agrobacterium*-mediated transformation.

Transformants were examined for the location of the T-DNA insertion and the proximity of the enhancers to annotated genes in the maize genome that were reported to be expressed at moderate levels in leaf tissues [44]. Determination of the site of integration of the construct was attempted for 223 events by a transgene border sequence identification method [45] and 107 of these events were mapped to locations in the maize B73 reference genome. To determine whether the enhancers within the T-DNA sequence are able to cause an increase in transcript accumulation of endogenous genes, these transformants were examined to identify T-DNA insertion sites within ~5.5 Kb of a gene. The CaMV 35S enhancer has been demonstrated to up-regulate genes within ~8 Kb of the enhancer sequences [29,46].

**Figure 7:**
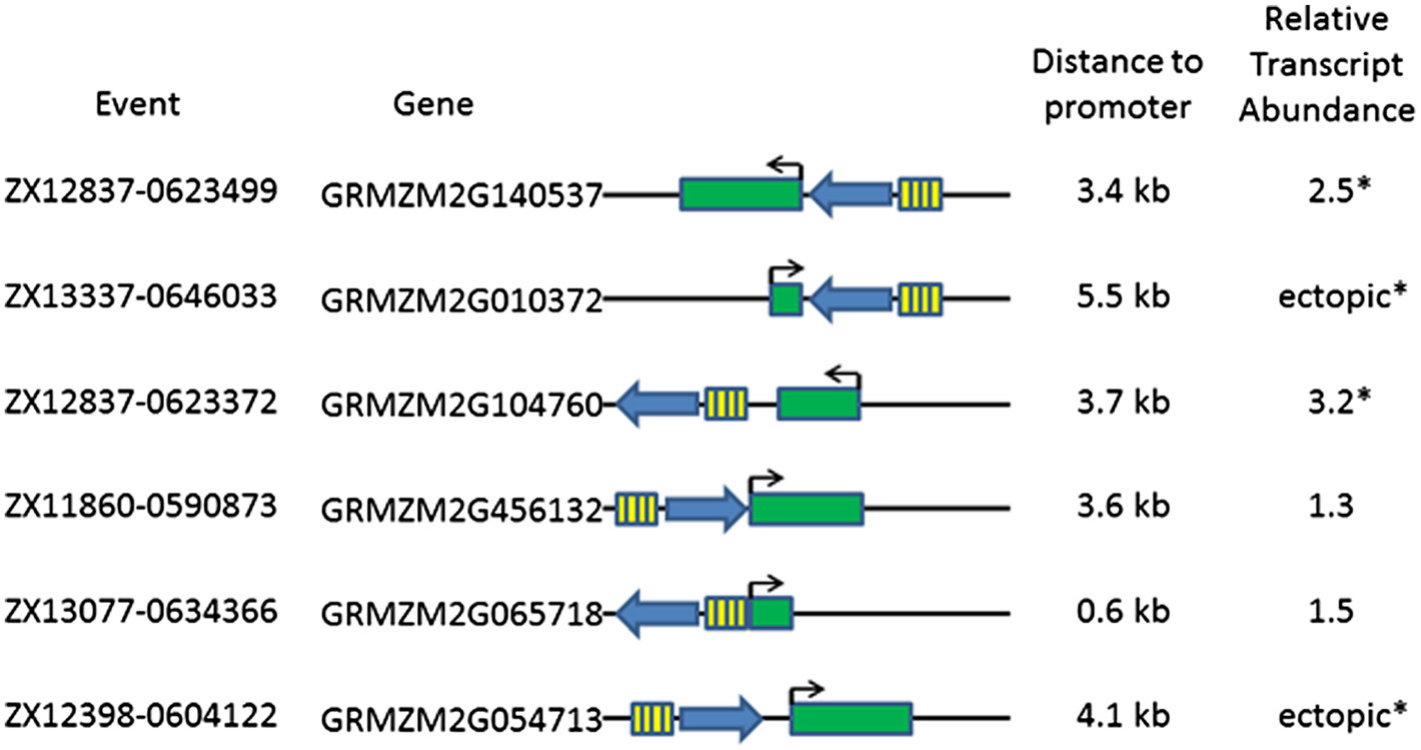
**Analysis of enhancer activity in transgenic maize plants.** The relative locations of the activation tagging element and the gene examined are shown. The activation tagging element is represented by four yellow boxes representing the 4× SCBV enhancer tandem array and the blue arrow representing the AAD1 selectable marker cassette. Maize genes are represented by the green box with the transcription start site and direction represented by the arrow. Distance to promoter reflects the estimated distance from the enhancers to the translational start site of the gene. The relative transcript abundance is the amount of transcript of the gene in transgenic plants compared with the level of the transcript in non-transgenic plants. An asterix indicates that the values were significantly different as determined by a t-test (α = 0.05).

Eleven events were examined to determine whether transcripts of genes adjacent to the activation tagging element were more abundant in the transgenic events than non-transgenic control plants. For five of these genes (GRMZM2G078472, GRMZM2G142119, GRMZM2G071986, GRMZM2G444075 and AC183888.4_FG008), no transcripts were detected in either the transgenic or non-transgenic lines. For the six other genes, transcripts were detected from genes adjacent to the activation tagging element. In these events, two genes (GRMZM2G456132 and GRMZM2G065718) showed a similar level of these transcripts in transgenic and non-transgenic plants (Figure 7). However, in 4 other events the transcripts of genes adjacent to the activation tagging element were more abundant in the transgenic plant than the non-transgenic plant. For two genes (GRMZM2G140537 and GRMZM2G104760), the transgenic plant showed more transcript than did the non-transgenic plant; this increase in abundance was 2.5 and 3.2 fold, respectively. For the other two genes (GRMZM2G010372 and GRMZM2G054713), no transcript was detectable in the non-transgenic plant but transcript was clearly detectable in the transgenic plant. These results demonstrate the 4x tandem array of the SCBV-IM enhancer can increase transcript abundance in a stable transformed maize plant and in some cases may cause ectopic expression of genes that are not expressed, or expressed at very low levels. It also indicates that the 4x tandem array of the SCBV-IM enhancer meets the traditional definition of an enhancer [47,48] because it can function upstream or downstream of the transcription unit and in either orientation.

## Discussion

The promoter sequences that we define include significant portions of the SCBV ORF III gene. The SCBV839 sequence, which has the greatest activity in transient assays, overlaps with 525 protein coding nucleotides. The sequences overlapping the ORF III gene contain most of the promoter activity as demonstrated by the SCBV333 fragment containing only 20 bp of the ORF III gene and having just 10% of the promoter activity of the SCBV839 sequence (Figure 3). The SCBV282 enhancer fragment contains 189 bp of ORF III coding sequence. A similar situation is found in Arabidopsis where regulatory elements for the promoter of *ZWICHEL* (*ZWI*) gene are found in exon and intron sequences of the adjacent *HYDROXYISOBUTYRL-CoA HYDROLASE 1* (*CHY1*) gene [49].

The enhancer sequences in the SCBV-IM promoter were able to increase the activity of the truncated ZmADH1 promoter (Figure 4), but these chimeric promoters were much weaker than the intact SCBV-IM promoter in the transient assays (Figure 3). This difference is so great it is unlikely to be the result of different cell preparations and may be the result of the SCBV-IM upstream activating sequences interacting differently with the heterologous core ZmADH1 promoter and the native SCBV-IM promoter. Similar results were seen when the CaMV 35S enhancer was placed upstream of the CaMV 19S core promoter [38].

Deletion analysis of the SCBV-IM promoter showed that removing sequences from −770 to −507 caused a 30% decline in promoter activity, while removing sequences from −770 to −264 caused a 90% decline in activity (Figure 3). It was, therefore, somewhat surprising to observe that the chimeric promoter SCBV537::ZmADH1 (containing SCBV-IM sequences −689 to −153) had less activity than the chimeric promoter SCBV282::ZmADH1 (containing SCBV-IM sequences −434 to −153) since the longer fragment in the promoter deletion analysis had the most activity. Surprising results are often obtained when portions of promoters are added or deleted and even small portions of promoters can have dramatic effects. For example, Dey and Matti [15] showed that removing 50 bp of the MMV promoter increased activity 10 fold and Simon et al. [50] showed that deleting 54 bp of the *Inner No Outer* promoter of Arabidopsis could reverse a silenced promoter.

Multiple copies of the SCBV-IM enhancer cause an increase in activity of the chimeric promoters in transient assays (Figure 5). This is consistent with what has been observed with the CaMV 35S and FMV ehancers [8,37-39] and may be due to multiple copies of the enhancer being more efficent in recruiting transcription factors to the promoter.

The SCBV282 fragment is capable of acting as a transcriptional enhancer when present in the maize genome in a 4x tandem array. In events containing the 4x SCBV-IM enhancer upstream and downstream of genes, and in either orientation with respect to these genes, increased transcript accumulation was observed (Figure 7). Furthermore, the element appeared to cause accumulation of transcripts that are not present, or present in very low levels, in non-transgenic lines. This demonstrates that the SCBV-IM enhancer may be used for activation tagging in maize. The SCBV-IM enhancers increased expression of 2 out of 8 genes that showed non-detectable expression in non-transgenic control plants. This is similar some studies that have reported ectopic expression of genes when the CaMV 35S enhancers integrate nearby [51,52].

## Conclusions

In this work, we demonstrate that the SCBV-IM promoter is comparable in strength to the ZmUBI1 promoter in transgenic young maize leaves and roots and we identify sequences from the SCBV-IM promoter that can function as a transcriptional enhancer in maize plants. We used transient assays to identify promoter sequences that are responsible for most of the promoter activity and sequences of this promoter that enhance expression from a heterologous promoter. Finally, we generated stable transgenic plants containing 4x tandem arrays of the SCBV-IM enhancer and demonstrated that transcripts of genes near the insertion site are more abundant than in non-transgenic control plants.

Activation tagging by randomly inserting transcriptional enhancers in the genome is a powerful tool for identifying gene function. The CaMV 35S enhancer has been used to develop activation tagging systems for Arabidopsis, rice and barley. Using these activation tagging systems, researchers have identified a number of genes with novel functions [28,53-57]. To date, no activation tagging system has been developed for maize. As a first step in developing an activation tagging system for maize, we have identified a transcriptional enhancer from SCBV-IM and have shown it to be able to activate transcription from a truncated ZmADH1 promoter in transient assays and from endogenous promoters in transformed maize plants.

## Methods

### 5′ RACE

Leaf tissue was collected from seedlings of transgenic event 625–1 containing the SCBV-IM::AAD1 construct. Total RNA was prepared using NucleoSpin RNA Plant kit (Macherey-Nagel, Ref. 740949). 5′ RACE was performed with AAD1 gene specific primer (GACTTGGTCTTTCTTCCACCTCACA) and SMARTer RACE 5′/3′ kit (Clontech Labratories, CA. Cat# 634858) following the manufacture’s recommended methods. Sequeneces generated from the 5′ RACE were then aligned to the reference sequences of SCBV-IM promoter and AAD1 gene to determine the transcription start site.

### Plasmid construction

The 839 bp SCBV-IM promoter sequence was synthesized by DNA2.0, Inc. The sequence is shown in Figure 2 (from GenBank accession AJ277091).

Two plant transformation vectors were constructed in the superbinary precursor plasmid pSB11. One of these contained the SCBV-IM promoter, aryloxyalkanoate dioxigenase herbicide resistance gene (AAD1) [40] and the maize Per5 3′ UTR, while the other contained the maize ubiquitin promoter [26], AAD1 and the maize Per5 3′ UTR. Between the T-DNA borders, these constructs also contained a ZmUBI promoter fused to the Phi Yellow Fluorescent Protein (PhiYFP) gene (Evrogen JSC, Moscow, Russia). These constructs were introduced into *Agrobacterium tumefaciens* strain LBA4404(pSB1) [58,59] to produce pDAB108625 and pDAB102110, respectively.

Fragments derived from the SCBV-IM promoter containing sequences −770 bp - +69 bp (plasmid pSCBV839), −507 - +69 bp (plasmid pSCBV576), and −264 - +69 bp (plasmid pSCBV333) of the SCBV-IM promoter were generated by PCR using the primers listed in Table 1. Fragments from the SCBV-IM promoter were cloned upstream of coding sequences for a firefly luciferase (LUC) reporter protein [60] (pEPP1020). The nopaline synthase (Nos) 3′ UTR region (bases 1847 to 2103 of GenBank Accession No. V00087.1) was cloned downstream of the LUC reporter gene to serve as a 3′ UTR.

**Table 1:** **PCR primers used to amplify portions of the SCBV promoter**

**Plasmid**	**Primer**	**Sequence**
pSCBV839	Forward	TCCCCGCGGAAGCTTATTGAATGGGGAAAACA
	Reverse	ACGCGTCGACTGCGGAAAGGTGTAATTCTTATTATTCAA
pSCBV576	Forward	TCCCCGCGGGGTTGAAAACTTCGACAAGAAAGCA
	Reverse	ACGCGTCGACTGCGGAAAGGTGTAATTCTTATTATTCAA
pSCBV333	Forward	TCCCCGCGGCCAGTGGAGGAGATCGTAAGCAATGA
	Reverse	ACGCGTCGACTGCGGAAAGGTGTAATTCTTATTATTCAA

Putative SCBV-IM enhancer sequences (−434 to −153, SCBV282 and −689 to −153, SCBV537) were PCR amplified from the SCBV-IM promoter region. Chimeric promoters were made by fusing enhancer fragments from the SCBV-IM promoter and a truncated promoter fragment from the maize alcohol dehydrogenase 1 (ZmADH1) gene corresponding to positions from −100 to +106 relative to the transcription start site [41]. The ZmADH1 promoter fragment was PCR amplified using genomic DNA from B73 using CGGGATCCGTATACCCACAGGCGGCCAAACCGC and CATGCCATGGTGCCCCCCTCCGCAAATCTT as the forward and reverse primers, respectively. The amplified PCR products were cloned upstream of the truncated ZmADH1 promoter fused to the luciferase gene. The promoter fragment was confirmed by sequencing. Two differences from the B73 reference sequence were observed; an “A” instead of a “G” at +44 bp and addition of “T” at residue +67 bp.

The 1x, 2x and 4x enhancer fragments of SCBV282 fragment were cloned in the BamHI and BstZ17I sites of pEPP1024, a plasmid containing the truncated ZmADH1 promoter fused to the LUC gene, for transient testing of the transcriptional enhancing activities. The 4x SCBV enhancer array was cloned into pSB11-derived plasmid pDAB3878 which also contains the rice actin1 gene promoter [61] driving the AAD1 selectable marker [40]. Superbinary constructs were then constructed by *in vivo* recombination of pSB1 plasmid and the newly constructed pSB11 derivative plasmid in recombinant *Agrobacterium tumefacians* strain LBA4404/pSB1 to form superbinary construct pEPS1027.

### Plant transformation

Constructs were introduced into the maize inbred line B104 using *Agrobacterium*-mediated transformation based on the superbinary method of Ishida et al. [62]. Maize plants (inbred B104) were grown in a greenhouse on a 16:8 hour Light:Dark photoperiod and hand pollinated using pollen from sibling plants. Immature embryos were isolated at 10 to 13 days after pollination when the embryos were 1.4 to 2.0 mm in size.

A suspension of *Agrobacterium* cells containing the superbinary vector pEPS1027 was prepared by transferring 1 or 2 loops of bacteria grown to solid medium containing 50 mg/L Spectinomycin, 10 mg/L Rifampicin, and 50 mg/L Streptomycin at 28° for 3 days and then a loop of this culture was used to innoculate 5 mL of liquid infection medium (MS salts, ISU Modified MS Vitamin stock (1000x, 2 g/L glycine, 0.5 g/L each of thiamine HCl and pyridoxine HCl, 0.05 g/L nicotinic acid, 3.3 mg/L Dicamba, 68.4 gm/L sucrose, 36 gm/L glucose, 700 mg/L L-proline, pH 5.2) containing 100 μM acetosyringone for 4 days at 25°C. This infection suspension was gently pipetted up and down using a sterile 5 mL pipette until a uniform suspension was achieved, and the concentration was adjusted to an optical density of 0.3 to 0.5 at 600 nm.

Prior to embryo excision and transformation, maize ears were surface sterilized. Immature embryos were then isolated and placed in 2 mL of infection medium. The medium was removed and replaced twice with 1 to 2 mL of fresh infection medium, which was then removed and replaced with 1.5 mL of the infection suspension and incubated for 5 minutes at room temperature. Then embryos were then transferred to co-cultivation medium and inubated for 3–4 day at 25°C in the dark. Co-cultivation medium contained MS salts, ISU Modified MS Vitamins, 3.3 mg/L Dicamba, 30 gm/L sucrose, 700 mg/L L-proline, 100 mg/L myo-inositol, 100 mg/L Casein Enzymatic Hydrolysate, 15 mg/L AgNO3, 100 μM acetosyringone, and 2.3 to 3 gm/L Gelzan™ (Sigma-Aldrich, St. Louis, MO), at pH 5.8.

After co-cultivation, the embryos were transferred to a MS-based resting medium containing MS salts, ISU Modified MS Vitamins, 3.3 mg/L Dicamba, 30 gm/L sucrose, 700 mg/L L-proline, 100 mg/L myo-inositol, 100 mg/L Casein Enzymatic Hydrolysate, 15 mg/L AgNO_3_, 0.5 gm/L MES (2-(N-morpholino)ethanesulfonic acid monohydrate; Fischer Scientific, Waltham, MA), 250 mg/L Carbenicillin, and 2.3 gm/L Gelzan™, at pH 5.8. Incubation continued for 7 days at 28°C in the dark. Following the 7 day resting period, the embryos were transferred to selection medium. MS-based resting medium (above) was used supplemented with Haloxyfop. The embryos were first transferred to selection medium containing 100 nM Haloxyfop and incubated at 28°C for 1 to 2 weeks, and then transferred to selection medium containing 500 nM Haloxyfop and incubated for an additional 2 to 4 weeks in the light (approximately 50 μEm^−2^ s^−1^). Transformed isolates were obtained in 5 to 8 weeks.

Following selection, cultures were transferred to an MS-based pre-regeneration medium containing MS salts, ISU Modified MS Vitamins, 45 gm/L sucrose, 350 mg/L L-proline, 100 mg/L myo-inositol, 50 mg/L Casein Enzymatic Hydrolysate, 1 mg/L AgNO_3_, 0.25 gm/L MES, 0.5 mg/L naphthaleneacetic acid, 2.5 mg/L abscisic acid, 1 mg/L 6-benzylaminopurine, 250 mg/L Carbenicillin, 2.5 gm/L Gelzan™, and 500 nM Haloxyfop, at pH 5.8 and incubated for 7 days at 28° under 24-hour white fluorescent light (approximately 50 μEm^−2^ s^−1^).

For regeneration, the cultures were transferred to an MS-based primary regeneration medium containing MS salts, ISU Modified MS Vitamins, 60 gm/L sucrose, 100 mg/L myo-inositol, 125 mg/L Carbenicillin, 2.5 gm/L Gelzan™, and 500 nM Haloxyfop, at pH 5.8 for 2 weeks at 28° in 24-hour white fluorescent light (approximately 50 μEm^−2^ s^−1^). Cultures were then transferred to an MS-based secondary regeneration medium composed of MS salts, ISU Modified MS Vitamins, 30 gm/L sucrose, 100 mg/L myo-inositol, 3 gm/L Gelzan™, at pH 5.8, with 500 nM Haloxyfop and regeneration continued for 2 weeks at 28°C under either 16-hour or 24-hour white fluorescent light conditions (approximately 50 μEm^−2^ s^−1^). When regenerated plants reached 3 to 5 cm in length, they were excised and transferred to secondary regeneration medium (as above, but without Haloxyfop) and incubated at 25° under 16-hour white fluorescent light conditions (approximately 50 μEm^−2^ s^−1^) to allow for further growth and development of the shoot and roots.

Regenerated plants were transplanted into Metro-Mix® 360 soilless growing medium (Sun Gro Horticulture) and placed a growth room. Plants were then transplanted into Sunshine Custom Blend 160 soil mixture and grown to flowering in the greenhouse. Controlled pollinations for seed production were conducted. In all cases, primary transformants were crossed with non-transformed B104.

### Transcript accumulation in transgenic plants

Transgenic plants were identified by a quantitative PCR assay of the AAD1 gene. Approximately 30 mg of T1 tissue was harvested from each of the tissues. Tissue samples were maintained on ice until placed at 4°C for storage until processing for DNA extraction. DNA was purified using the BioSprint DNA 96 plant kit following the manufacturer’s instructions (Qiagen cat. No. 941558). Samples were normalized to 5 ng/μL for qPCR template. A Picogreen assay (Invitrogen, cat No. P11496) was performed to quantify DNA.

For transcript accumulation assays, samples were collected from leaves, roots and tassels at different times during development. For leaves, samples were collected at the V3 growth stage (14 days after planting) from the 3rd fully expanded leaf, at the V8 growth stage (41 days after planting) from the 8th fully expanded leaf and at the R1 growth stage (71 days after planting) from the leaf just below the ear. Samples from the root were collected at the V3 (14 days after planting) and V10 (51 days after planting) growth stages; one cm samples were collected from the tip of a root. Tassels were collected at the R1 growth stage by sampling an entire branch of the tassel. First strand cDNA was synthesized following manufacturer’s instructions using the High Capacity cDNA synthesis kit (Invitrogen, cat No. 4368813) in a 10 μL reaction containing 5 μL of total RNA. Following synthesis, cDNA was diluted 1:3 with nuclease free water. Quantitative PCR assays were set up using the Eppendorf epMotion5075 liquid handler. Each sample was assayed in triplicate for target gene (AAD1) and a reference gene TIP (GRMZM2G095185) for leaf and tassel tissues or MAZ95 (GRMZM2G053299) for root tissues. Each well contained 4 μL of assay mix (Roche Universal Probe Library (UPL)) and 1 μL of cDNA was added. Reference assay mix consisted of forward (AGCCAAGCCAGTGGTACTTC) and reverse (TCGCAGACAAAGTAGCAAATGT) primer at a final concentration of 0.25 μM and UPL probe at a final concentration of 0.1 μM with 1x Light Cycler480® Probes Master mix. AAD1 assay mix consisted of forward (AACCATGCAAGCCACCAT) and reverse (GGTAGAGGGAACCGAACACA) primer at a final concentration of 0.375 μM and UPL probe #53 at a final concentration of 0.1 μM with 1x Light Cycler480® Probes Master mix.

Detection was 6FAM channel in both assays. PCR cycling conditions were initially activated at 95°C for 10 minutes followed by 43 cycles of denaturation at 95°C for 10 seconds, annealing and extension at 60°C for 20 seconds and data acquisition for 1 second at 72°C. Assay plates were run on the Roche LC480II and analysis performed by relative quantification.

### Transient assays in maize suspension cultures

Maize Hi-II suspension culture cells [63] were transfected by particle bombardment with plasmid DNA constructs harboring promoter or enhancer elements driving the LUC gene and a control plasmid DNA construct containing a ZmUBI1::GUS gene for normalization of transfection.

Bulk preparations of plasmid DNAs were prepared using QiAfilter™ Plasmid Maxi Kits (Qiagen, Germantown, Maryland) and the quantity and quality were analyzed using standard molecular methods. The Hi-II cells were grown by shaking at 125 rpm in H9CP+ medium (H9CP medium consists of MS salts 4.3 gm/L, sucrose 3%, Casamino acids 200 mg/L, myo-inositol 100 mg/L, 2,4-D 2 mg/L, NAA 2 mg/L, 1000X MS vitamins 1 mL/L, L-proline 700 mg/L, and coconut water (Sigma Aldrich, St. Louis, MO) 62.5 mL/L, pH 6.0) at 28°C in the dark. Prior to bombardment, the 2-day old Hi-II cultures were transferred to G-N6 medium (CHU N6 medium 3.98 g/L, CHU N6 vitamins 1 mL/L (both CHU components were from PhytoTechnology Laboratories®, Lenexa, KS), Myo-inositol 100 mg/L, 2,4-D 2 mg/L and sucrose 3%, pH 6.0) and allowed to grow for 24 hours. On the day of bombardment, 2.5 g of G-N6 grown cells were transferred to sterile Whatman No. 1 filter disks (55 mm) placed on G-N6 medium containing 0.5 M D-sorbitol and 0.5 M D-mannitol and incubated for 4 hours. The osmotically-adjusted cells were used for bombardment.

Gold particles (1 μm diameter, BioRad, Hercules, CA) were washed with 70% ethanol for 10 minutes, then three times with sterile water. The particles were dispensed in 50% glycerol at a concentration of 120 mg/mL. For a typical experiment, 150 μL (18 mg) of gold particles, approximately 5 μg of plasmid DNA, 150 μL of 2.5 M CaCl_2_ and 30 μL 0.2 M spermidine were combined. The reaction (total volume 375 μL) was incubated at room temperature for 10 minutes with occasional gentle vortexing. The DNA coated-gold particles were briefly centrifuged, washed with 420 μL of 70% ethanol and then with 420 μL of 100% ethanol. The final pellet was resuspended in 110 μL of 100% ethanol and subjected to a brief sonication (three bursts of 3 seconds each, with 1 minute between bursts) with a Branson 1450 sonicator.

Aliquots of 12.2 μL of the gold-particles coated with DNA were spread on each of nine macrocarriers (BioRad, Hercules, CA) and used in bombardment assays using a BioRad PDS1000/He system. The suspension culture cells were transfected at a target distance of 9 cm using 3510 psi disks and each plate was bombarded 3 times. Following bombardment, the cells were incubated in the dark at 28°, first for 12 hours on G-N6 containing D-sorbitol and D-mannitol medium, then on G-N6 plates for an additional 36 hours. Cells were collected from the plates, blotted to remove buffer and extracted with 300 μL of 2x CCLT LUC extraction buffer (Promega Corporation, Madison, WI). After centrifugation, about 600 μL of protein extract was collected. Protein concentrations were estimated using the Bradford assay.

LUC enzymatic activity (expressed in Luciferase Units (LU)/mg protein) and GUS enzymatic activity (expressed in GUS activity units (GU)/μg protein) were measured as previously described [64]. Relative activities of the test promoters in SCBV:LUC constructs were compared by normalizing LUC levels to GUS levels as the ratio of LUC/mg protein:GUS/μg protein.

### Analysis of activation tagging events

Flanking sequence from left border of the T-DNA insert for each line transformed with pEPS1027 was determined by sequencing PCR products derived using a transgene border sequence identification method [45]. Mapping and identification of distances to nearest genes upstream and downstream of the insertion was performed by an automated flanking sequence analysis program [65]. The location of the insert was verified by PCR using left border flank primer, SHnstF (CTGTTCCTGACTATGCTGGCAAGT), as forward primer paired with a locus specific reverse primer as indicated in Table 2.

**Table 2:** **Primers for insertion site mapping confirmation**

**Name**	**Locus**	**Sequence**
EA56	GRMZM2G140537	GATCTTTTCTGGGGAGCGGTTC
EA200	GRMZM2G010372	ATAGAACGGAGGTGTCCAAAGTCTC
EA191	GRMZM2G104760	GCTCGTTTTTTCCCCCATAGC
EA7	GRMZM2G456132	ACACCTTGCCGCACCGC
EA65	GRMZM2G065718	GGGTACTAGCTCAATCGTCGCTC
EA45	GRMZM2G054713	AGAGTTTACTCATGCCGCAGCC

To determine whether transcripts of genes adjacent to the activation tagging element were more abundant in transgenic plants than non-transgenic plants, leaf samples were taken from V5 leaf tissues [44] and total RNA isolated as before. Transcript abundance was measured using quantitative reverse transcriptase PCR (RT-qPCR). First strand cDNA was prepared using the high capacity cDNA Reverse Transcription kit (Life Technologies #4368814) in a 10 μL reaction volume with 250–500 ng total RNA. Reaction products were diluted 1:3 with water and assayed using the Absolute Blue qPCR SYBR Green kit (ThermoFisher #AB-4166B). PCR reactions containing each primer at 200 nM final concentration and 1 μL of diluted template in a 7 μL final volume were performed. Primers used in gene specific assays are shown in Table 3. The PCR program consisted of activation at 95°C for 15 minutes followed by cycling with sequential steps of denaturation at 95°C for 15 seconds, annealing at 58°C for 30 seconds and extension at 72°C for 15 seconds with the last step being used for data acquisition. A total of 40 cycles were used.

**Table 3:** **PCR primer information for gene expression assays**

**Name**	**Sequence**	**Assay locus**
U1LT01_139_L	CTCGTGGAAGTCGGTGAAG	GRMZM2G140537
U1LT01_139_R	ATCAGCTTGGACATCTCCTG	
U4LT01_443_L	GTTGCGTGGCGAGTAACAT	GRMZM2G010372
U4LT01_443_R	GACGACATTCATGGCAGTTG	
U6RT01_272_L	CGAGTCGAAAGAAACGCTTG	GRMZM2G104760
U6RT01_272_R	ATATATCGCAACTCACGCCC	
U7RT01_640_L	GGTTATTTCACCGCTCACGA	GRMZM2G456132
U7RT01_640_R	TTTGTTCATGTCCCATGACG	
U10RT01_150_L	CTTTCAAGTTCGCCATCCTC	GRMZM2G065718
U10RT01_150_R	GCCTCGTACGTCTTGAGCAC	
U14RT02_12_L	TCATTGAACGCTAGCTGCTG	GRMZM2G054713
U14RT02_12_R	AAAGCTGGGGTTGGAATTG	

### Availability of supporting data

The data sets supporting the results of this article are included within the article.
